# Supporting medicines management for older people at care transitions – a theory-based analysis of a systematic review of 24 interventions

**DOI:** 10.1186/s12913-021-06890-7

**Published:** 2021-08-30

**Authors:** Justine Tomlinson, Iuri Marques, Jonathan Silcock, Beth Fylan, Judith Dyson

**Affiliations:** 1 Medicines Optimisation Research Group, School of Pharmacy and Medical Sciences, University of Bradford, Bradford, UK; 2 Medicines Management and Pharmacy Services, Leeds Teaching Hospitals NHS Trust, Leeds, UK; 3 Bradford Institute for Health Research, Bradford Teaching Hospitals NHS Foundation Trust, Bradford, UK; 4 Faculty of Health, Education and Life Sciences, Birmingham City University, Birmingham, UK

**Keywords:** Behaviour change, Intervention, Medicines management, Older people, Theoretical domains framework, Systematic review

## Abstract

**Background:**

Older patients are at severe risk of harm from medicines following a hospital to home transition. Interventions aiming to support successful care transitions by improving medicines management have been implemented. This study aimed to explore which behavioural constructs have previously been targeted by interventions, which individual behaviour change techniques have been included, and which are yet to be trialled.

**Method:**

This study mapped the behaviour change techniques used in 24 randomised controlled trials to the Behaviour Change Technique Taxonomy. Once elicited, techniques were further mapped to the Theoretical Domains Framework to explore which determinants of behaviour change had been targeted, and what gaps, if any existed.

**Results:**

Common behaviour change techniques used were: goals and planning; feedback and monitoring; social support; instruction on behaviour performance; and prompts/cues. These may be valuable when combined in a complex intervention. Interventions mostly mapped to between eight and 10 domains of the Theoretical Domains Framework. Environmental context and resources was an underrepresented domain, which should be considered within future interventions.

**Conclusion:**

This study has identified behaviour change techniques that could be valuable when combined within a complex intervention aiming to support post-discharge medicines management for older people. Whilst many interventions mapped to eight or more determinants of behaviour change, as identified within the Theoretical Domains Framework, careful assessment of the barriers to behaviour change should be conducted prior to intervention design to ensure all appropriate domains are targeted.

## Background

Harm from medication is estimated to cost the global economy $42 billion USD annually [1] and causes significant distress and burden to patients [2, 3]. Medication safety has thus been identified as an international priority; the World Health Organization (WHO) tasks countries to halve severe medicines-related harm by 2022 [2]. The accompanying WHO strategic framework highlights that the systems and practices of medication (prescribing, dispensing, administration and monitoring) are one of four main domains to target [4].

Medication-related errors and other issues linked to medicines management (i.e. non-adherence to regimen, experience of side effects, etc.) can occur at multiple stages of the patient pathway; though their likelihood is increased during high-risk situations. It is known, for example, that 2–3% of primary care encounters and 10% of hospital encounters result in medication error [5]. High-risk situations tend to be categorised by complex combinations of processes, technology and human interaction [6]. One such risky situation for medication safety occurs at transitions of care [2]. A “transition of care” is a broad term describing the transfer of a patient’s care from one health professional and/or setting to another [7]. Medication discrepancy, which is an unintentional mismatch between two medication lists, is one example of medication error that is prevalent at transitions. It is reported that between 30 and 70% of patients experience an unintended medication discrepancy when their care is transferred between settings, such as during hospital discharge [8]. Additionally, between 11 and 59% of medication discrepancies at admission and discharge have been reported as potentially harmful [6]. Medication safety has thus been identified as one of the core components in successful transitions of care, alongside educating patients to self-manage and advance care planning [9].

Some patient groups are also at higher risk of harm from medicines at transitions. For example, older patients who often take multiple medicines are likely to have several changes made to their medicines during inpatient admission. This increases the risk of harm from medicines after hospital discharge [10] if patients are not fully aware of changes or their primary healthcare team are not informed, or do not act on changes. A study found that one in three older people (*n* = 413/1116; 37%) in the UK experiences medicines-related harm in the 8 weeks after discharge; 3.4% (*n* = 14/413 cases) of which is due to medication error and 10.9% (*n* = 49/413 cases) due to non-adherence to medications [11]. There is considerable economic impact surrounding medication errors, such as, the healthcare resources to treat patients that have experienced an error or the time taken by clinicians to resolve any medication-related problems and legal proceedings [6]. Ensuring appropriate medication management, the processes and behaviours that support safe and effective medication use, is thus a priority, especially for older patients in the post-discharge phase.

Strategies for promoting successful transitions through medication management have been suggested, including improving engagement with patients, medicines reconciliation and better information transfer between care settings [12–14]. Increasing shared decision making and encouraging meaningful conversations around medicines has been suggested by the Department of Health and Social Care in the UK [15], as one priority to overcome medicines errors. In the UK, the National Health Service (NHS) Long Term Plan [16] acknowledges the historical divide between care settings and suggests new models of care, such as integrated care systems, which aim to promote joined up care. Whilst these strategies have been identified as possible solutions to increase medicines safety at transitions, there is limited guidance on how to implement them. To enact safe medicines management at hospital discharge and in the post-discharge phase, it is important to understand how discrete behaviours contribute to positive patient outcomes [17]. It is also vital to explore possible techniques to promote positive behaviour change amongst patients and healthcare professionals to increase the safety of medicines management at transitions of care [18].

According to Michie et al. [19], complex interventions that aim to alter behaviour have historically been poorly designed because they are underpinned by personal experience, favoured theory or cursory analysis, rather than scientific evidence. It is, therefore, posited that developing an understanding of the likely mechanisms of behaviour change is a crucial step in complex intervention design [17]. Hence, research is needed to explore the mechanisms of action of these effective interventions. This is particularly important for interventions that support post-discharge medicines management for older people. Retrospective mapping using an appropriate theoretical framework is one method that has proved effective previously [20]. The Behaviour Change Technique Taxonomy (BCTT v1) [21] is an example of a framework that can be used to characterise ‘active’ ingredients of existing interventions. Compiled by expert consensus, to standardise the reporting of the content of interventions, it is an exhaustive list of 93 discrete behaviour change techniques (BCT), grouped in 16 clusters, based on their definitions [21]. For example, the BCTs problem solving and action planning are grouped under ‘Goals and Planning’. Applying this taxonomy to trialled behaviour change interventions, in order to characterise their content, and then applying the resultant techniques to a framework, such as the Theoretical Domains Framework (TDF), allows for comprehensive, systematic and coherent analysis [18]. The TDF provides a list of 14 (originally 12, Version 1) theoretical constructs relevant to behaviour change determinants, identified from 33 psychological theories and validated by consensus [22, 23]. These are: knowledge, skills, social/professional identity and role, beliefs about capabilities, beliefs about consequences, motivation and goals, memory, attention and decision processes, environmental context and resources, social influences, emotion, action planning and nature of behaviours. Each domain is associated with component constructs that aid the researcher to consider the cognitive, affective, social and environmental influences on behaviour [24].

Through mapping intervention components to the BCTT and TDF, we can explore which behavioural constructs have previously been targeted by interventions, which individual BCTs have been included, and which are yet to be trialled. Interventions that are tailored are more effective than those that are not [25]; by considering existing interventions in the light of the contextual literature relating to behavioural determinants/barriers and facilitators to safe medicines management following a hospital to home transition we can suggest intervention components that are likeliest to be effective. The findings from this theory-based analysis will therefore help inform future intervention design.

### Aim and objectives

This study is part of a wider programme of research investigating interventions to support successful hospital to home transitions for older people through medication management. A systematic review of the interventions found in existing literature has been published previously [14]. The aim of this additional study was to conduct a secondary analysis of these interventions that support either patient or healthcare professional behaviours to (a) investigate the possible theoretical underpinning of interventions previously identified and (b) explore potential mechanisms of behaviour change to enhance medication management after discharge. The specific objectives were to: identify specific behaviour techniques for each intervention component guided by the Behavioural Change Techniques Taxonomy v1 [21], and pinpoint the most commonly used techniques as well as possible gaps; and map behaviour techniques to the Theoretical Domains Framework v1 [22] to identify which of the possible behavioural determinants are being targeted and which are not i.e. the likely mechanisms of action.

## Methods

A literature review of reported interventions to enhance medicines management at hospital discharge was conducted. To promote rigour and transparency, the Preferred Reporting Items for Systematic Reviews and Meta-analyses (PRISMA) checklist was used in the systematic review and it was registered [PROSPERO (CRD42018086873)]. Full information, including the literature search strategy and quality appraisal, is provided in the open-access publication [14]. Briefly, it comprised the systematic identification in published literature of randomised controlled trials from 2003 to 2019 from various databases, written in English and reporting care transition (hospital to home) interventions, that support post-discharge medicines management, for patients aged 65 and older. A theory-based analysis was then conducted by three authors, underpinned by the BCTT and the TDF V1.

### Behaviour change technique taxonomy (BCTT)

Initially, behavioural components were identified for each intervention and mapped to the BCTT in an iterative process. Firstly, three of the authors from different professional backgrounds and experience (i.e. elderly care pharmacist (JT), psychologist (IM) and behaviour change expert (JD)), independently mapped each intervention component to the BCTT, using published definitions and examples [18, 21, 26]. BCTs were coded for two recipients of the interventions where applicable: patients and healthcare professionals. The authors then met to discuss individual mappings and identify and resolve discrepancies. After this first meeting, two of the authors met at five different points in time to reach a consensus on all the BCTs for each component. A final meeting was held with all members of the team to discuss any discrepancies and agree on the final mapping.

### Theoretical domains framework (TDF)

Following the mapping of each component to the BCTT, the resultant BCTs were further mapped to eleven domains of the original twelve domain TDF (v1) that were likely to have been targeted by BCTs. The domain “nature of behaviour” was excluded as this domain supports the description and definition of the target behaviour rather than consideration of the factors that determine the behaviour. We chose to use the original 12 domain version of the TDF as this has the most extensive mapping to the BCTT providing a clearer basis on which to make decisions, and published expert consensus on the BCTs likely to influence each TDF domain was used [18]. For those BCTs that were not listed or had not been linked to the TDF, a more recently published consensus exercise was considered [26]. Finally, for any remaining BCTs identified which had not or could not be classified, the authors met to discuss and arrive a consensus, drawing on their psychological and clinical knowledge and professional expertise of healthcare practice in the NHS in the UK (for example medicines reconciliation, when it is performed and how it is routinely conducted). The final results were agreed by all of the authors.

## Results

The study characteristics for each intervention have been described elsewhere [14]. Briefly, 24 interventions were found from 12 different countries; the majority from Europe (*n* = 11) followed by the USA (*n* = 6), Australia (*n* = 2), Canada (*n* = 2), Singapore (*n* = 2) and Taiwan (*n* = 1) (see Table 1). Some interventions were provided during hospital admission (*n* = 9) [27–35], others provided in the post-discharge period (*n* = 6) [36–41] and the remainder were commenced in hospital but continued after discharge, effectively bridging the transition (*n* = 9) [42–50]. Studies used varying numbers and combinations of components within interventions and were most often delivered by pharmacists (*n* = 13) or nurses (*n* = 5).

**Table 1:** Study characteristics

Study details	Participants and setting	Intervention components
**Interventions commenced during hospital admission**		
Basger et al. [31]	216 elderly patients admitted to a small private hospital, taking ≥5 medicines, Australia	Medication counselling, Medicines reconciliation, Medication review to detect drug related problems, Self-management discussions, Information transfer
Bolas et al. [27]	162 patients admitted for unplanned causes to the medical admissions unit, taking ≥3 long term medicines, Northern Ireland	Preparation of full medication history, Medicines reconciliation, Patient education and discharge counselling, Pharmaceutical discharge letter, Personalised medicines record sheet, Medicines helpline
Graabaek et al. [34]	400 patients, admitted to the medical acute unit, Denmark	Structured medication review, Medicines reconciliation, Recommendations for change reported to clinician, Medication report created to aid clinician preparing discharge, Patient counselling
Hockly et al. [33]	33 patients, taking ≥4 medicines, UK	Information transfer
Lalonde et al. [29]	83 patients, being discharged with ≥2 medicines changes, Canada	Medication Discharge Plan created and given to patient at discharge, Transfer of information to Primary Care Provider and Community Pharmacist by fax
Legrain et al. [30]	665 patients, admitted to the acute geriatric unit with stays longer than 5 days, France	Comprehensive chronic medication review, Medicines reconciliation, Patient education and self-management discussion, Transition of care communication with outpatient healthcare professionals
Scullin et al. [28]	762 elderly patients, admitted to medical wards, taking ≥4 long term medicines OR one high risk medicines OR previous admission within last 6 months OR given an IV antibiotic on day one of admission, Northern Ireland	Medicines reconciliation, Medication review, Counselling, Medicines record sheet, Information transfer
Tamblyn et al. [35]	4656 patients, discharged from internal medicine, cardiac or thoracic surgery units, Canada	Electronic medicines reconciliation, Information transfer
Tong et al. [32]	832 patients, admitted to general medical unit at an adult major referral hospital, Australia	Personalised medication management plan
**Interventions commenced at hospital admission and continued post-discharge**		
Buurman et al. [48]	674 elderly patients, admitted to the internal medicine ward, Netherlands	Medicines reconciliation, Discussion with Primary Care Provider and additional support enabled, Home visit for patient education
Casas et al. [43]	155 patients with COPD and minimum admission length of 48 h in two tertiary hospitals, Belgium and Spain	Educational programme (2 h) on self-management, Information transfer, Post-discharge telephone calls, Web-based call centre
Chan et al. [46]	699 patients, admitted to internal medicines, family medicines, cardiology or neurology wards at a general safety net hospital and trauma centre, USA	Patient education, Self-management coaching, Medicines reconciliation, Written medicines information, Post-discharge telephone calls, Medicines helpline
Coleman et al. [49]	750 elderly patients, with a long-term condition, with admission to large hospital/ service delivery system, USA	Personalised patient-held record, Home visit for education, Self-management coaching, Medicines reconciliation, Post-discharge telephone calls
Gillespie et al. [44]	400 elderly patients (> 80 years) admitted to two internal medicines wards at a University Hospital, Sweden	Medicines reconciliation, Medication review, Patient education, Information transfer, Post-discharge telephone call
Huang and Liang [42]	126 elderly patients, admitted to large medical hospital with hip fracture due to falling, Taiwan	Individualised discharge plan, Information brochure, Patient education, Home visit, Post-discharge telephone calls, Medicines helpline, Collaboration with Primary Care Provider
Koehler et al. [45]	41 elderly patients, taking ≥5 long term medicines and with ≥3 chronic conditions, admitted to a University Hospital, USA	Pharmacist-led medicines reconciliation, Medication review, Patient education including self-management, Post-discharge telephone call, Personal health record, Information transfer
Lee et al. [47]	840 patients, admitted to medical ward of tertiary hospital and at high risk of readmission, Singapore	Patient education, Medicines reconciliation, Medication review, Discharge information, Post-discharge telephone calls, Home visit
Ravn-Nielsen et al. [50]	974 patients, taking ≥5 medicines, admitted to the acute admission wards, Denmark	Structured medication review, Information transfer, Medicines reconciliation, 30-min motivational interview with patient at discharge for education and self-management, Post-discharge telephone calls
**Interventions commenced post-discharge**		
Ahmad et al. [37]	340 elderly patients, taking ≥5 long term medicines, discharged from general or academic hospitals, Netherlands	Medication review, Medication counselling using cognitive behaviour techniques, Home visit, Medicines reconciliation, Collaboration with Primary Care Provider, Removal of redundant medications from home
Char et al. [40]	200 patients, taking ≥5 long term medicines, attending first outpatient clinic appointment following recent stay in hospital, Singapore	Medicines reconciliation, Collaboration with Primary Care Provider, Best possible medication history created for patient
Gurwitz et al. [38]	3661 elderly patients, discharged from hospital for any admission, USA	Information transfer, System prompt to schedule an appointment within one week
Haag et al. [39]	25 elderly patients, discharged from tertiary care academic medical centre for any type of admission, USA	Post-discharge telephone call, Medication review, Medicines reconciliation, Information transfer
Holland et al. [36]	872 elderly patients, from 10 hospitals following an emergency admission and taking ≥2 medicines, UK	Home visit, Medication review, Patient education, Collaboration with primary care provider, Removal of redundant medications from home
Tuttle et al. [41]	159 patients, discharged from large tertiary-referral hospital following acute illness and detection of chronic kidney disease stage 3–5, USA	Home visit, Medicines reconciliation, Medication review, Patient education and self-management strategies, Information transfer

Each BCT identified in this theory-based analysis (see Table 2) was attributed to healthcare professionals, patients or both, depending on who was responsible for receiving the intervention. An example of a healthcare professional behaviour targeted by a component was the accuracy checking of medicines when a patient is admitted to hospital. An example of a patient behaviour was contacting a clinician after discharge if they needed advice. An example of a behaviour involving both healthcare professionals and patients was involvement and engagement in action planning (such as in goal setting).

**Table 2:** BCTs coded within interventions

	Hospital only interventions																		Bridging interventions																		Post-discharge interventions											
	Basger et al. [31]		Bolas et al. [27]		Graabaek et al. [34]		Hockly et al. [33]		Lalonde et al. [29]		Legrain et al. [30]		Scullin et al. [28]		Tamblyn et al. [35]		Tong et al. [32]		Buurman et al. [48]		Casas et al. [43]		Chan et al. [46]		Coleman et al. [49]		Gillespie et al. [44]		Huang and Liang [42]		Koehler et al. [45]		Lee et al. [47]		Ravn-Nielsen et al. [50]		Ahmad et al. [37]		Char et al. [40]		Gurwitz et al. [38]		Haag et al. [39]		Holland et al. [36]		Tuttle et al. [41]	
BCT identified*	PT	HCP	PT	HCP	PT	HCP	PT	HCP	PT	HCP	PT	HCP	PT	HCP	PT	HCP	PT	HCP	PT	HCP	PT	HCP	PT	HCP	PT	HCP	PT	HCP	PT	HCP	PT	HCP	PT	HCP	PT	HCP	PT	HCP	PT	HCP	PT	HCP	PT	HCP	PT	HCP	PT	HCP
1.1 Goal setting (behaviour)	√		√		√				√		√		√				√		√		√	√	√		√		√		√		√		√		√		√		√						√		√	
1.2 Problem solving		√	√	√		√				√		√		√		√				√	√	√	√	√	√		√	√	√	√	√	√	√	√	√	√	√	√		√				√	√		√	
1.3 Goal setting (outcome)											√			√							√		√		√				√		√	√	√		√		√								√			
1.4 Action planning		√	√	√		√		√	√	√	√	√		√			√	√	√	√	√	√	√	√	√		√	√	√	√	√	√	√	√	√	√	√	√		√		√		√	√	√	√	√
1.5 Review behaviour goal(s)		√		√		√				√				√		√							√	√	√		√	√	√		√	√	√		√	√	√			√			√		√		√	
1.6 Discrepancy between current behaviour and goal		√		√	√	√				√	√	√	√	√		√			√	√	√		√	√	√		√	√	√		√	√	√	√	√	√	√	√		√				√	√		√	
1.7 Review outcome goal(s)																				√											√			√	√		√								√		√	
1.8 Behavioural contract											√																																					
1.9 Commitment																																			√		√											
2.2 Feedback on behaviour					√						√		√								√		√		√		√		√		√		√		√		√						√		√		√	
2.3 Self-monitoring of behaviour	√										√								√		√		√		√				√		√		√		√		√						√		√		√	
2.4 Self-monitoring of outcome(s) of behaviour	√										√								√		√		√		√				√		√		√		√		√						√		√		√	
2.5 Monitoring outcome of behaviour without feedback														√																		√		√														
2.7 Feedback on outcome(s) of behaviour		√		√		√		√		√		√		√				√										√				√			√	√	√					√		√		√	√	√
3.1 Social support unspecified	√		√		√								√				√				√		√		√		√		√		√		√		√								√		√		√	
3.2 Social support (practical)						√				√	√			√					√	√	√	√		√	√			√	√	√	√				√	√	√	√		√					√			
3.3 Social support (emotional)											√										√		√		√										√		√											
4.1 Instruction on how to perform behaviour	√		√		√				√		√		√				√		√		√	√	√		√		√		√		√		√		√		√		√				√		√		√	
4.2 Information on antecedents																							√						√						√		√											
4.3 Reattribution																							√												√		√											
4.4 Behavioural experiments																							√												√		√											
5.1 Information about health consequences	√		√		√						√		√						√		√		√		√		√		√		√		√		√		√						√		√		√	
5.2 Salience of consequences													√																						√		√											
5.3 Information about social and environmental consequences																																			√		√											
5.4 Monitoring of emotional consequences																							√												√		√											
5.5 Anticipated regret																							√												√		√											
5.6 Information about emotional consequences																							√												√		√											
6.1 Demonstration of behaviour																									√																						√	
7.1 Prompts/ cues		√	√	√		√		√	√	√		√	√	√			√	√			√	√	√	√	√		√	√	√	√	√	√	√		√	√	√	√	√	√		√		√	√	√		√
7.7 Exposure																							√												√		√											
8.1 Behavioural practice/ rehersal																					√		√		√				√		√		√		√		√								√			
8.2 Behaviour substitution																							√												√		√											
8.3 Habit formation																							√												√		√											
8.4 Habit reversal																							√												√		√											
8.6 Generalisation of target behaviour																							√												√		√											
9.1 Credible source	√		√	√					√				√				√				√		√		√		√		√		√		√		√		√		√									
9.2 Pros and cons						√								√									√											√	√	√	√											
9.3 Comparative imagining of future outcomes																							√												√		√											
10.4 Social reward																																			√		√											
10.9 Self-reward																							√																									
11.2 Reduce negative emotions																							√												√		√											
11.3 Conserving mental resources																							√												√		√											
12.1 Restructuring the physical environment													√										√												√		√								√		√	
12.2 Restructuring the social environment																							√												√		√										√	
15.1 Verbal persuasion about capability	√										√								√		√		√		√		√		√		√		√		√		√								√		√	
15.2 Mental rehersal of successful performance																			√				√		√										√		√											
15.3 Focus on past successes																			√				√		√						√				√		√											
15.4 Self-talk																							√												√		√											
16.2 Imaginary reward																																			√		√											
Total number of individual BCTs	14		11		13		3		10		16		18		3		7		13		17		39		21		14		18		21		19		45		44		9		3		12		19		18	

*PT = BCT directed toward patient behaviour

HCP = BCT directed toward healthcare professional behaviour

### Behaviour change techniques linked to medicines management at transitions

The studies that used the most discrete BCTs were Ravn-Nielsen et al. (*n* = 45) [50], Ahmad et al. (*n* = 44) [37] and Chan et al. (*n* = 39) [46], the majority of which targeted patient behaviours. All three of these interventions made use of motivational interviewing, a multifaceted behaviour change technique, which accounts for the large number of BCTs. In contrast, the interventions that used the least number of techniques were Hockly et al. (*n* = 3) [33], Tamblyn et al. (*n* = 3) [35], Gurwitz et al. (*n* = 3) [38], Tong et al. (*n* = 7) [32] and Char et al. (*n* = 9) [40]. These tended to focus solely on information transfer between care providers [32, 33, 38] or medicines reconciliation [35, 40].

The results presented below start by describing the most and least popular BCT groupings (those with the largest or least number of BCTs reported within the group). Subsequently, the most and least reported individual techniques will be described. This means that whilst a grouping may not have been popular, certain techniques may feature in many, if not the majority, of interventions. A brief example is the grouping *Associations*, which is one of the least popular groupings as only two out of a possible eight techniques have been reported. However, one of the individual techniques (prompts and cues) is used by 22 of the 24 interventions.

The most widely used BCT grouping was *Goals and Planning*. This group contains techniques focused on setting, reviewing and solving issues around goals. Within this grouping, the majority of components were aimed at patient behaviours (*n* = 49), followed by those where both patient and healthcare professional behaviours were targeted (*n* = 40). The groupings *Feedback and Monitoring* (ongoing monitoring and review of behaviours and outcomes) and *Social Support* (emotional, practical or unspecified) were also well represented within the interventions. Intervention components that focused on feedback and monitoring targeted primarily patient behaviours, with only two studies focusing on both behaviours [41, 50]. Ravn-Nielsen et al.’s intervention [50] offered the patient feedback on outcomes of behaviour during a 30-min motivational interview at discharge, which included education and self-management coaching. They also communicated outcomes verbally to the healthcare professional when problems were identified and transferred information to the primary care provider. Tuttle et al. [41] similarly provided feedback on outcomes to the prescriber and offered support to patients by reviewing their self-management strategies.

Those interventions that focused on healthcare professional behaviours used predominantly feedback on outcomes of behaviour, most often by transferring discharge information between care providers to alert them to medication changes, prompting action [27, 31, 44, 46]. Three interventions monitored outcomes without offering feedback to the patient [28, 45, 47]. This was most often via medication review where monitoring of therapeutic goals was conducted, without patient involvement. For those studies focusing on patient behaviours, the majority used feedback on the behaviour (*n* = 15), self-monitoring of the behaviour (*n* = 14) and self-monitoring of outcome (*n* = 14).

Although a large number of interventions mentioned techniques linked to social support, the majority (*n* = 16) did not specify what support was provided. In nine interventions the social support specified was practical and in six interventions the support was emotional [30, 37, 43, 46, 49, 50]. For those coded as practical support, six targeted healthcare professional behaviours, four targeted patient behaviours and five targeted both. Examples of practical support for professionals included collaboration between hospital clinicians and General Practitioners [42], and hospital pharmacies communicating with community pharmacies to resolve prescription issues [46]. Examples of practical support for patients included self-management educational programmes [43], adherence support [37] and home visits that promoted self-management [49]. For components coded as emotional support, all helped patients cope with medicines management after hospital discharge. Examples included empowerment [43] and coaching techniques [46] for self-management at home.

The least popular groupings, reported only by two interventions each were *Comparison of Behaviour* (learning by performing the behaviour or seeing how behaviour should be performed) [41, 49] and *Covert Learning* (encouraging behaviour through imaging reward, punishment and consequences) [37, 50]. These targeted patient behaviours only. An example of *Comparison of Behaviour* is rehearsal or role-play of intended behaviour [49]. An example of *Covert Learning* is motivational interviewing [50]. There were two groupings that did not feature in any of the interventions identified: *Identity* and *Scheduled Consequences*.

Although some groupings were not used frequently in the identified interventions, individual BCTs within these groupings were popular across the majority of studies. Four well utilised techniques were:

- Instruction on how to perform the behaviour (belonging to the grouping *Shaping Knowledge),* for example the component patient and carer education [36];
- Information about health consequences (belonging to the grouping *Natural Consequences),* for example the component motivational interview at hospital discharge [50];
- Prompts and cues (belonging to the grouping *Associations),* for example the component individual care plan shared across care teams [43]; and
- Credible source (belonging to the grouping *Comparison of Outcomes),* for example component personalised medication record sheet containing instructions for the patient [28].

The remainder of the techniques were used less frequently within the studies (see Table 2).

### Theoretical domains linked to post-discharge medication management

Six interventions included components that encompassed all domains of the TDF [28, 36, 37, 41, 46, 50] (see Table 3). Most of the others utilised between eight and 10 of the domains. The three least complex interventions focused only on healthcare professional behaviours [33, 35, 38] and targeted the fewest domains (four to five domains). All three of these interventions were electronic based and served to transfer information [33, 38] or highlight medicines reconciliation issues [35].

**Table 3:**
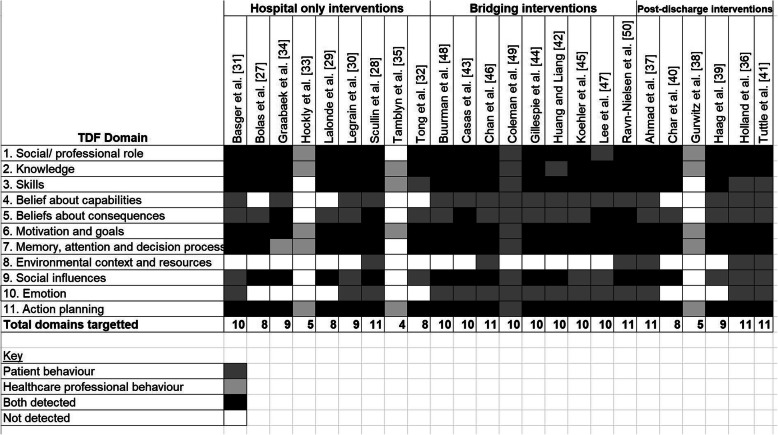
TDF domains coded within interventions

*Motivation and Goals, Action Planning* and *Knowledge* were the only domains that were prevalent within all studies. *Social and Professional Role* and *Memory, Attention and Decision Processes* featured in 23 of the 24 interventions. The least utilised domain was *Environmental Context and Resources*, with only six interventions incorporating this [28, 36, 37, 41, 46, 50]. *Emotion (n = 15 studies)* was the second least represented domain.

## Discussion

This study has identified the common BCTs and target theoretical domains of 24 interventions that aimed to support post-discharge medicines management for older people. This has allowed an exploration of the potential mechanisms of behaviour change of these trialled interventions to enhance medicines management after discharge. Analysis of findings showed that the most prevalent BCT groupings were *Goals and Planning, Feedback and Monitoring* and *Social Support*. These are valuable aspects of medication management, for example, defined plans for adherence, monitoring and follow-up, who should be involved when, how and why, and what support the patient may need, should be in place. Some groupings, although rarely used, had a number of BCTs that were prevalent across interventions. Individual BCTs included: instruction on how to perform the behaviour (*Shaping Knowledge*) or prompts and cues (*Associations*). Although the groupings were not popular, the individual techniques are core components of patient education about how to manage medicines after a hospital stay and are therefore important to any intervention of this kind. For example, in order for patients to successfully manage their medicines after discharge, it is crucial that they are given specific instructions about which medicines to take, how and when.

Common determinants of post-discharge medicines management include: disruption in the patients’ medicine knowledge base and routines caused by hospitalisation (likely to be mapped to TDF domains action planning, knowledge), knowledge gaps and the need for new information [51, 52] (knowledge), the need to develop new routines [51, 53] (action planning), the need for temporary practical support [51–53] (beliefs about capabilities), whether or not a person considered managing medicines their own responsibility [51] (role and identity), and the relationship between healthcare professionals and patient [52]. The included interventions addressed many of these. For example, knowledge gaps was considered in all but three interventions through “instruction on how to perform the behaviour” (exceptions: [33, 35, 38]). Disruption to and the need to develop new routines was addressed through a range of strategies with all but four studies using goal setting (exceptions: [33, 35, 38, 39]. Less frequently used strategies to support routines included action planning [49], contracting [30] and prompts or cues [47, 49]. The need for support was addressed by the majority (exceptions: [29, 33, 35, 38, 40, 48]. The need to promote the patient as having shared responsibility for their medicines was potentially through interventions that included social support, persuasion about capability and mental rehearsal of successful performance, one of which was included in all interventions except six (exceptions: [29, 30, 33, 35, 38, 40]).

As discussed above some of the interventions included *many* intervention components/BCTs. Whilst doing so does ensure that a wide range of behavioural determinants are addressed and there is strong evidence that multifaceted interventions are effective [54], this can be an expensive approach and lead to wasted effort and resources. Tailoring to determinants/need is therefore both effective and economical [25]. Equally, some of our included studies had interventions including very few components which may not have addressed key determinants. The interventions with the fewest BCTs were aimed at supporting provider *clinical* behaviour rather than patient behaviour; these were restricted to prompts/cues, action planning and feedback [33, 38] or problem solving, reviewing and identifying discrepancies in behaviours and goals [35]. Key barriers for healthcare professionals include lack of time and low self-efficacy [55], not addressed by the BCTs proposed by Hockly et al. [33], Tamblyn et al. [35] and Gurwitz et al. [38].

A small number of interventions focused solely on tasks aimed at healthcare professionals, suggesting lack of patient involvement and therefore limited exploration of patients’ individual needs. Considering the patient goes home often with complex regimens, it is vital that patients are involved in discussion and decisions about their medicines [56]. Hence it is likely that the interventions that only focus on healthcare professional behaviours would have benefitted from involving patients and their carers at some point in the intervention. To this effect, the majority of interventions offered components focused on both patient and healthcare professional behaviours, further strengthening the argument that targeting patient behaviours is crucial to the success of any intervention aimed at medicines management. Putting the patient at the centre of their care is likely to result in better outcomes [57, 58]. Based on the findings, it is clear that priority has been given to patient behaviours in most of the interventions in recognition that patients should have an active role in their care. However, what remains unclear is how these BCTs were implemented and consequently whether behaviours were adopted effectively, and what the extent of the patient involvement was, since the interventions descriptions within the studies were lacking in specific detail. Our findings suggest any future intervention should focus on harnessing individual patient strategies and formalise them to ensure effective management, even when the patients have returned home.

Interventions targeting patient behaviours were predominantly prescriptive (such as providing written or verbal information, rather than working with them in partnership). Only a very small number of interventions involved the patient significantly (for example through role play, counselling and motivational interviewing) through strategies which can highlight potential issues before they happen and address any concerns that patients may have. However, even these interventions did not offer sufficient detail as to what activities entailed, such as in the case of ‘counselling’. In pharmacy practice and research, counselling is a term frequently used to describe a brief medicines consultation or the provision of specific advice on medicines use. This is not extended or person-centred counselling that might be provided by a therapist or specialist nurse. It is unclear, however, from the interventions described what the term ‘counselling’ meant, which not only makes it difficult to replicate these components, but the extent to which these interventions helped the patient cannot be ascertained.

It is also unclear whether pharmacists had the relevant skills to deliver in-depth counselling or motivational interviewing interventions. Whilst the UK pharmacy curriculum includes relevant learning outcomes related to the demonstration of effective consultation skills and working with patients to decide a course of action [59], it is not at all clear that this means defining problems from the patient’s perspective (as true counselling would) or identifying personal goals (as motivational interviewing would). Whilst there is evidence to support the effectiveness of motivational interviewing for medicines management [60] and this has influenced modern undergraduate curricula; it remains unclear if these skills are widespread or utilised effectively without further professional development [61, 62]. Many studies mentioned healthcare professional training but they did not describe the content and depth of this training [30, 31, 39, 41, 44, 45, 48]. Ravn-Nielsen et al. [50] and Ahmad et al. [37] provided a medication review workshop and motivational interviewing course for their study pharmacists. These courses were delivered over two or days three and it is unknown whether this was a suitable course length for becoming proficient in motivational interviewing. Difficulties identifying and replicating essential core components of interventions is an established challenge to other practitioners who wish to utilise them. Despite guidance calling for an improvement in the completeness of reporting for interventions [63], the studies within this review did not allow for detailed descriptions of what, why, when and how for each component to be identified.

All interventions had components spanning the TDF. Only one domain was underrepresented: *Environmental Context and Resources*. Interventions that mapped to this domain, had components that involved seeing patients in their own homes and included the removal of old medicines or product standardisation whilst in hospital. Whilst a lesser reported barrier than those cited above, this was identified in the literature [51–53]. Therefore, it might prove useful for any intervention that includes home visits to remove unnecessary medicines as that will not only reduce confusion and complexity but will inherently help the patient reconcile it in their own minds. Barriers to medicines reconciliation for patients and healthcare professionals were generally not reported in the included papers. Similarly environmental barriers such as time pressures or lack of human resource (clinical staff, formal or informal carers) have been identified in the literature [55, 64–66] but not included in interventions. For example, a study of primary care practitioners (doctors, nurses and pharmacists) identified environmental issues including time as the second greatest barrier to safe prescribing for frail patients [67].

Additionally, *Emotion* was poorly represented throughout the studies which is perhaps surprising since medication management can be an emotional experience for patients [56]. One possible explanation for the lack of BCTs linked to emotion could be that the barriers to behaviour change were not fully considered during intervention development. Without assessment of these barriers and behavioural determinants prior to intervention design, it is difficult to effectively target the mechanism of action for behaviour change within the local context. Furthermore, unless a theoretical approach is used to assess barriers, cognitive bias may lead to failure to fully recognise or report barriers [68]. For these reasons, it is imperative to use a theoretical approach to assessing barriers and designing interventions.

One of the striking findings was that the outcomes investigated were rarely behaviour focused. Rather, the majority of interventions focused on outcomes related to error, harm and health in general, and did not appear to link these desired outcomes with the behaviours that would help these to be achieved. Whilst these clinical outcomes are important, recording changes in behaviour following implementation of new interventions, would allow for the investigation of whether the intervention was effective in helping patients cope with their medication management across the pathway, particularly at transitions, an area still under researched to date. Similarly, very few interventions stated that they were underpinned by a theory of behaviour change (*n* = 4; McKeeham and Coulton’s discharge plan model [42]; theory of planned behaviour [37]; conceptual framework of integrated practice units [47]; 5As model of behaviour change [41]), an essential tenet of complex intervention design [17]. Consequently, the majority of these interventions were task rather than behaviour driven. Many of the interventions within this study included a medicines reconciliation component, for example. Whilst literature pertaining to the barriers to effective medicines reconciliation, from the perspective of healthcare professionals [55] and patients [69] exists, they are mainly task orientated. Barriers such as limited resource, inaccurate tools or unclear information [55] do not lend themselves readily to BCTs without deeper examination of the underpinning behavioural determinants. Although the majority of interventions within this study did not explicitly aim to change behaviour, it is likely that they facilitated change due to the nature of some components, such as motivational interviewing and counselling. Future interventions should therefore also focus on the measurement of efficiency and efficacy linked to behaviour change.

For purposes of the analysis, every intervention was broken into its individual components. However, it is important to acknowledge that although every component was analysed individually, their effects cannot be taken in isolation. When considered individually, it might be a weak point but in the context of the full intervention and in interaction with the other components, clinical effectiveness might be greater. In other words, the effectiveness of the intervention is greater than the sum of the effectiveness of each component.

Finally, complex intervention design guidance illustrates the importance of involving patients and other key stakeholders in the co-design of such interventions [17, 70]. Only three studies [28, 30, 45] documented having designed the studies with key stakeholders (for example healthcare professionals), however none explicitly mentioned patient or public involvement. In the UK there is a drive to involve patients at every stage of healthcare, including design and development of interventions. Patients are generally willing to and want to be involved in decisions that affect their care, and having the opportunity to participate in co-design can highlight important patient behaviours, goals, priorities and concerns that would otherwise remain undetected [70].

### Strengths and limitations

A strength of this study is linked to the use of multiple sources of information to map intervention components to BCTT and the TDF. Specifically, analysis drew from the established literature on the BCTs and TDF, the authors’ multidisciplinary expertise and experience, and relevant local and national healthcare policies and guidelines. This was particularly useful to counteract the limitation linked to a lack of detail about crucial components in each intervention. Because many of the interventions lacked clarity about specific behaviours in each component, the authors made a number of coding assumptions based on their own professional research and experience as well as policies and guidelines. For example, where interventions described medication review, the authors assumed a number of steps expected to ensure a comprehensive review of medicines. Another example of this pertains to interventions where motivation interviewing is described as a component. Although it was not possible to ascertain what motivational interviewing entailed, experience and expertise in the field of Psychology and healthcare allowed for agreement amongst the authors regarding a number of assumptions made, when coding these components to the BCTT and TDF. Additionally, several iterative discussions were held to ensure a consensus was reached for every single intervention component. To further strengthen analysis, study authors were contacted where clarification was needed, although many did not respond. For this reason, and despite the authors’ efforts, it is possible that interpretation for each component may have overestimated the number of steps expected compared to what really may have happened in each intervention.

To ensure uniformity, the authors also made every attempt to standardise analysis for every intervention. To accomplish this, common components were identified across all interventions to ensure they were analysed in the same way. For example, for all interventions where medicines reconciliation took place, the authors assumed that this component involved the same BCTs and was performed to the same quality expected, unless explicitly stated otherwise.

Finally, in some instances, mapping intervention components to the BCTT was not straightforward and some components were not a perfect fit. In such instances, this limitation was overcome by initially using different literature sources [18, 22, 26]. Subsequently, the authors met at different occasions to reach a consensus, again drawing from their own experience and expertise in research, psychology and healthcare in the UK.

## Conclusions

This theory-based analysis has identified certain BCT groupings and discrete BCTs that are common amongst studies aiming to support successful care transitions through medication management. We offer insights for the development of a novel intervention that incorporates those BCTs with potential impact, but also those that appear underutilised. *Goals and Planning, Feedback and Monitoring* and *Social Support,* along with instruction of how to perform the behaviour and prompts/ cues are elements that could be valuable when combined within a complex intervention. Whilst many interventions mapped to eight or more determinants of behaviour change, as identified within the TDF, careful assessment of the barriers to behaviour change should be conducted in the first instance to ensure all appropriate domains are targeted. Environmental context and resources was an underrepresented domain and should be considered within future interventions.

## Data Availability

The datasets during and/or analysed during the current study available from the corresponding author on reasonable request.

## Abbreviations

BCT: Behaviour Change Technique
BCTT: Behaviour Change Technique Taxonomy
NHS: National Health Service
TDF: Theoretical Domains Framework
UK: United Kingdom
WHO: The World Health Organisation
